# MRgFUS of small breast cancer: what should be learned from a case of local recurrence

**DOI:** 10.1186/2050-5736-3-S1-O75

**Published:** 2015-06-30

**Authors:** Hidemi Furusawa, Junichi Shidooka, Masuko Inomata, Emiko Hirabara, Hiroshi Nakahara, Yukiko Ymaguchi

**Affiliations:** 1 Breastopia Clinic, Miyazaki, Japan

## Background/introduction

Background: The aim of local treatment of breast cancer is to completely eradicate the cancer cells. The excisionless study which is an alternative local treatment for the breast cancer with MRgFUS is going on at our facility from June 2005. Purpose: To validate the safety and efficacy of this local treatment. And to probe the cause of local recurrence.

## Methods

The main inclusion criteria; 1) breast cancer diagnosed by the needle biopsy 2) Receptor status confirmation 3) tumor size 15mm 4) well demarcated mass in contrast enhanced MRI. The main exclusion criteria; 1) pure type mucinous carcinoma 2) invasive micropapillary carcinoma 3) location of the tumor requires a high sonication angle. The postoperative needle biopsy was performed again within three weeks after ablation and no residual viable cancer cells were identified pathologically. The following radiotherapy should be performed. The patients are followed by three modalities every 3 to 6 months.

## Results and conclusions

Eighty-seven patients were enrolled and 72 breast lesions were treated sequentially. The median age was 56 years old (29 - 79). The average tumor size was 11.0mm (5 - 15). The average treatment duration was 126 minutes (41-246). The median follow-up period was 68 months (2-108). Thirty-eight cases have been followed up for more than 60 months. Although there were no severe adverse events and any distant recurrence cases, the local recurrence developed seven years after the initial treatment in only one invasive breast cancer case. There are two presumptive causes of this local recurrence. The one reason could be the cancer displacement by the preoperative needle biopsy and another one may be the developing residual cancer after MRgFUS, according to the post MRgFUS needle biopsy specimen and the pathological inspection after excision. Conclusions: MRgFUS as a local treatment for small breast cancer has the potential of replacing usual surgery.

However the radio therapy obviously can control the local recurrence, the careful follow-up should be needed.

